# First series of da Vinci single-port robotic-assisted cervical oesophagectomy: single-centre IDEAL stage 2a/2b study

**DOI:** 10.1093/bjsopen/zrag030

**Published:** 2026-06-05

**Authors:** Vladimir J Lozanovski, Luca Bellaio, Edin Hadzijusufovic, Olga Adamenko Meier, Franziska Renger, Christoph Wandhoefer, Suzanne S Gisbertz, Richard van Hillegersberg, Hauke Lang, Peter P Grimminger

**Affiliations:** Department of General, Visceral and Transplant Surgery, University Medical Center of the Johannes Gutenberg-University Mainz, Mainz, Germany; Department of General, Visceral and Transplant Surgery, University Medical Center of the Johannes Gutenberg-University Mainz, Mainz, Germany; Department of General, Visceral and Transplant Surgery, University Medical Center of the Johannes Gutenberg-University Mainz, Mainz, Germany; Department of General, Visceral and Transplant Surgery, University Medical Center of the Johannes Gutenberg-University Mainz, Mainz, Germany; Department of General, Visceral and Transplant Surgery, University Medical Center of the Johannes Gutenberg-University Mainz, Mainz, Germany; Department of General, Visceral and Transplant Surgery, University Medical Center of the Johannes Gutenberg-University Mainz, Mainz, Germany; Department of Surgery, Amsterdam UMC Location University of Amsterdam, Amsterdam, the Netherlands; Cancer Treatment and Quality of Life, Cancer Center Amsterdam, Amsterdam, the Netherlands; Department of Surgery, University Medical Centre, Utrecht, the Netherlands; Department of General, Visceral and Transplant Surgery, University Medical Center of the Johannes Gutenberg-University Mainz, Mainz, Germany; Department of General, Visceral and Transplant Surgery, University Medical Center of the Johannes Gutenberg-University Mainz, Mainz, Germany

**Keywords:** minimally invasive oesophagectomy, robotics, oesophageal cancer surgery, inflatable mediastinoscopic, transcervical oesophagectomy

## Abstract

**Background:**

Recent advances in robotic technology, particularly the da Vinci single-port (SP) system, have improved transcervical mediastinoscopic access by minimizing robotic arm collisions in confined spaces.

**Methods:**

This study, based on IDEAL (Idea, Development, Exploration, Assessment, Long-term) framework, describes the first consecutive da Vinci SP robotic-assisted cervical oesophagectomy (RACE) procedures in patients with oesophageal carcinoma performed at the University Hospital Mainz. Patient demographics, neoadjuvant treatment, resection margins, lymph node yield, intraoperative parameters, postoperative recovery, complications, and mortality were assessed.

**Results:**

The study included 20 patients with primarily squamous cell carcinoma, with 70% of all patients having received neoadjuvant treatment. The surgical procedure demonstrated feasibility, evidenced by a 90% R0 resection rate and a mean(standard deviation) lymph node yield of 31(7). The mean(standard deviation) total operative time was 332(88) minutes, and the active console time was 91(48) minutes. Notably, there were no intraoperative complications or conversions. Patients experienced minimal postoperative pain, with no need for epidural or intercostal catheter analgesia; by postoperative day 5, all patients received analgesia on demand. Most patients began ambulating on the first postoperative day, and the median hospital length of stay was 8 (range 5–86) days. Postoperative complications included pneumonia in five patients and anastomotic leakage in four. Two patients died: one from fulminant pulmonary embolism and one from multiple organ failure secondary to sepsis following pneumonia and anastomotic insufficiency.

**Conclusion:**

The da Vinci SP system was successfully used for the first time in a clinical series of RACE procedures. SP RACE offers comparable operating times and lymph node retrieval to robotic transthoracic procedures, low postoperative pain and rapid recovery. However, the current scope of the SP RACE procedure is limited, and its outcomes warrant further prospective investigation to better define its role, refine patient selection, and evaluate long-term results.

## Teaser

This study presents the first clinical experience with single-port robotic-assisted cervical oesophagectomy (SP RACE) using the da Vinci SP system in 20 consecutive patients with oesophageal cancer. The procedure was technically feasible but was associated with substantial perioperative morbidity in a highly selected, co-morbid patient population. These early results underline the need for careful patient selection and further prospective studies to define the role and safety profile of SP RACE.

## Introduction

The multimodal standard approach to treating locally advanced oesophageal cancer includes either chemotherapy or chemoradiation followed by oesophagectomy^1,2^. Minimally invasive oesophagectomy (MIE) is superior to open surgery, with numerous studies indicating that robotic-assisted MIE (RAMIE) addresses the challenges posed by rigid thoracic wall, inflexible instruments, and limited manoeuvrability encountered during MIE^3–5^. However, some patients cannot tolerate the transthoracic approach due to co-morbidities or previous lung surgeries. To overcome this limitation, robotic-assisted cervical oesophagectomy (RACE) using the da Vinci Xi multiport and the da Vinci single-port (SP) prototype system (Intuitive Surgical, Sunnyvale, CA, USA) has been introduced. The RACE procedure was performed as a combined robotic-assisted transcervical (TC) and transabdominal approach, with the abdominal phase performed using a conventional multiport robotic system; this combined technique was termed TC-RAMIE-RACE^6–9^.

The recently introduced da Vinci SP system enables effective mediastinal dissection via a TC approach, owing to reduced collisions between robotic arms in narrow spaces such as the mediastinum and a magnified view of the operative field^10^. Due to the TC approach and the avoidance of thoracotomy, this technique may also reduce postoperative morbidity, particularly pulmonary complications, which are the most common complications after oesophageal surgery^11^. Nevertheless, a potential concern regarding the transmediastinal approach is the number of lymph nodes retrieved and the high rate of recurrent laryngeal nerve palsy^12^. To date, clinical evidence on the RACE procedure remains very limited and is restricted to small case series demonstrating technical feasibility rather than comparative efficacy or long-term oncological outcomes.

The innovative SP RACE procedure was implemented at the University Hospital Mainz in April 2024 as part of a structured introduction of this novel technique^10,12^. This study reports the initial institutional experience in a carefully selected patient cohort, including patients previously considered unsuitable for transthoracic oesophagectomy due to cardiopulmonary co-morbidities or an inaccessible right chest.

## Methods

This retrospective case series study was conducted in accordance with the IDEAL (Idea, Development, Exploration, Assessment, Long-term) framework for surgical innovation and STROBE guidelines for observational research, ensuring transparent and comprehensive reporting of the study design, data collection, analysis, and interpretation^13,14^. SP RACE was introduced and evaluated in a stepwise manner corresponding to stage 2a/2b of the IDEAL framework, with a focus on technical refinement, safety, feasibility, and early clinical outcomes.

The primary aim of this study was to evaluate the feasibility and short-term safety of SP RACE, focusing on perioperative morbidity. Secondary outcomes included postoperative pain, early mobilization, and overall recovery. Oncological parameters were analysed as descriptive feasibility measures rather than definitive oncological endpoints.

To reduce bias, all SP RACE procedures were performed by a consistent surgical team within a single institution, ensuring procedural standardization. Data were collected prospectively and transparently documented in an institutional database, with the inclusion of consecutive eligible patients to minimize selection bias. Systematic and comprehensive follow-up was conducted for all patients to ensure completeness of outcome assessment. Informed consent was obtained from all patients, allowing for the anonymous collection of clinical data and follow-up information for potential scientific use. According to applicable federal state regulations (§36 and §37 of the Rhineland-Palatinate state hospital law) and the guidance of the independent ethics committee (Ethics Committee of the State Medical Association of Rhineland-Palatinate), formal ethics approval was not required for this study.

### Patient characteristics and perioperative data

**Fig. 1:**
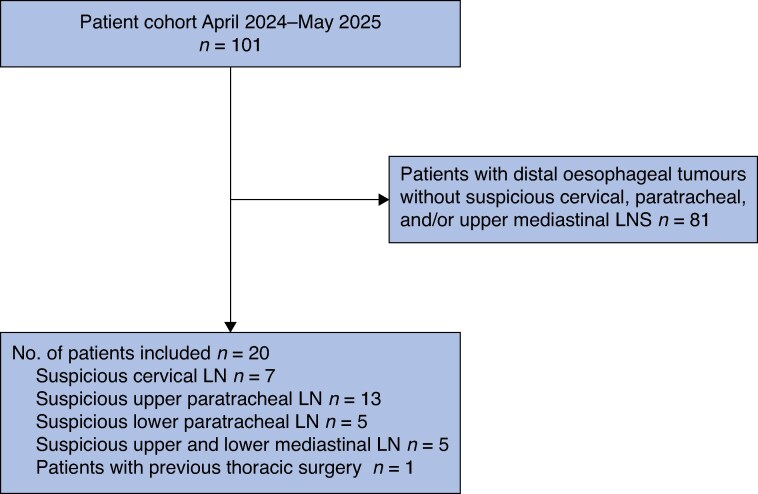
Flow chart of the patient selection process Numbers of patients meeting the inclusion criteria are shown. Individual patients may have fulfilled more than one inclusion criterion, so the total number exceeds 20. LN, lymph node.

Patients who underwent SP RACE were identified from a prospective institutional database, and data were extracted from both written and electronic medical records. The first 20 consecutive da Vinci SP RACE procedures performed on adult patients at University Hospital Mainz since April 2024 were analysed. All SP RACE procedures at the University Hospital Mainz were performed using the da Vinci SP System, along with its associated instruments. Patients were selected based on tumour location; those with tumours extending above the carinal bifurcation, suspiciously enlarged paratracheal and/or cervical lymph nodes, or co-morbidities such as previous lung surgery were considered for the SP RACE procedure. The standard diagnostic work-up consisted of cervico-thoraco-abdominal computed tomography (CT) scanning and gastroduodenoscopy with biopsy of the oesophageal tumour. Routine fine-needle aspiration was not performed before neoadjuvant treatment; however, in unclear cases, positron emission tomography–CT may have been recommended by the multidisciplinary tumour board. The patient selection process is shown in the flow chart (*Fig. 1*). Tumour location was categorized following the Japanese classification of oesophageal cancer^15^.

Demographic information was collected prospectively. Perioperative data were also gathered and analysed prospectively, including diagnosis, preoperative chemoradiotherapy, tumour node metastasis (TNM) stage, resection margin status (R), and the Charlson Comorbidity Index (CCI)^16^. Preoperative gastroscopy with endoscopic through-the-scope pyloric balloon dilation using Boston Scientific CRE™ through-the-scope balloons (balloon length 5.5 cm, diameter 20 mm, and pressure 20 mmHg) was performed in all patients with non-occlusive tumours. Additional variables included the total duration of the procedure, the length of the mediastinoscopic phase, active instrument time, the duration of stay in the intensive or intermediate care unit, and any readmissions to these units. Total procedure duration was defined as the time from abdominal skin incision until the completion of the cervical skin suture. The duration of the mediastinoscopic phase was measured from cervical skin incision to cervical skin suture. Active console time represents the cumulative period during which robotic instruments were engaged, reflecting the actual dissection and preparation performed by the surgeon at the console.

Postoperative pain was assessed using the visual analogue scale, and pain management strategies (peridural analgesia, paravertebral analgesia, patient-controlled analgesia, and on-demand analgesics) were prospectively recorded. Furthermore, total hospital length of stay (days), perioperative complications, intraoperative mortality, in-hospital mortality, and 30-day and 90-day mortality were documented. Perioperative complications were classified according to the Esophagectomy Complications Consensus Group^17^. Laryngoscopy was performed on all patients before and after the operation to assess for recurrent laryngeal nerve palsy. Perioperative data were analysed and compared between the first ten patients (Group A) and the second ten patients (Group B). All patients were routinely seen in the outpatient clinic after discharge, and follow-up was performed up until December 2025.

### Surgical procedure

**Fig. 2:**
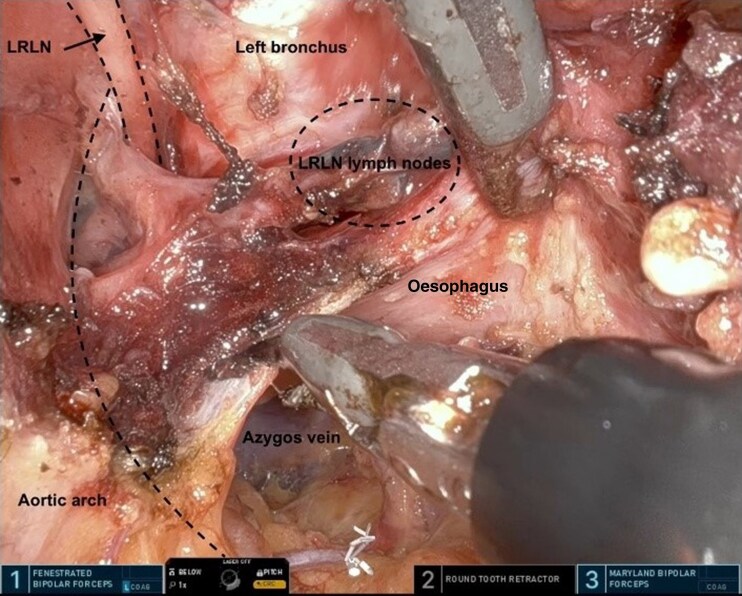
Visualization and dissection of the left LRLN LRLN, left recurrent laryngeal nerve. The dashed lines represent the course of the aortic arch, the LRLN, and the area of LRLN lymph nodes.

**Fig. 3:**
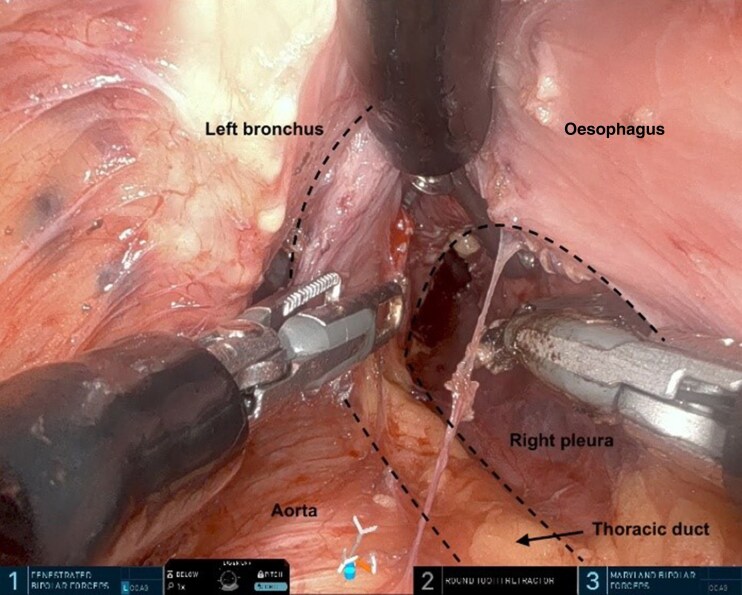
Visualization and dissection of the thoracic duct The dashed lines mark the thoracic duct and the infracarinal area.

**Fig. 4:**
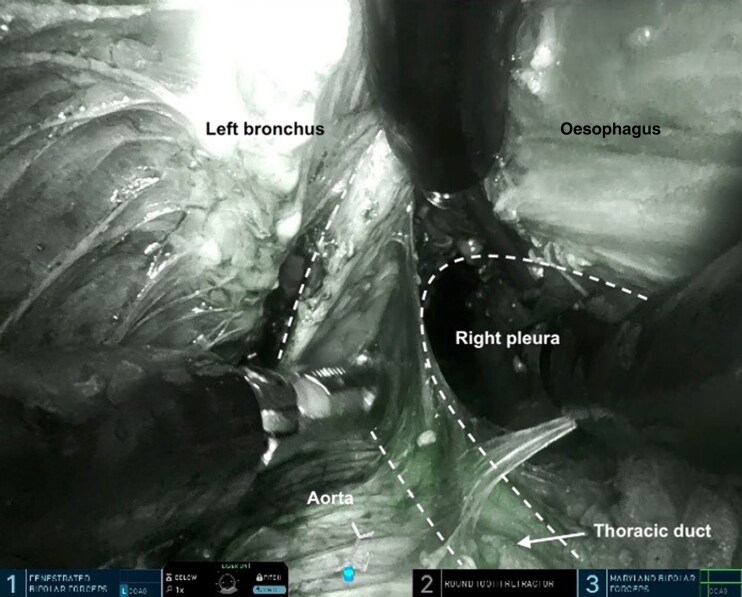
Visualization and dissection of the thoracic duct using indocyanine green in firefly mode The dashed lines mark the thoracic duct and the infracarinal area.

**Fig. 5:**
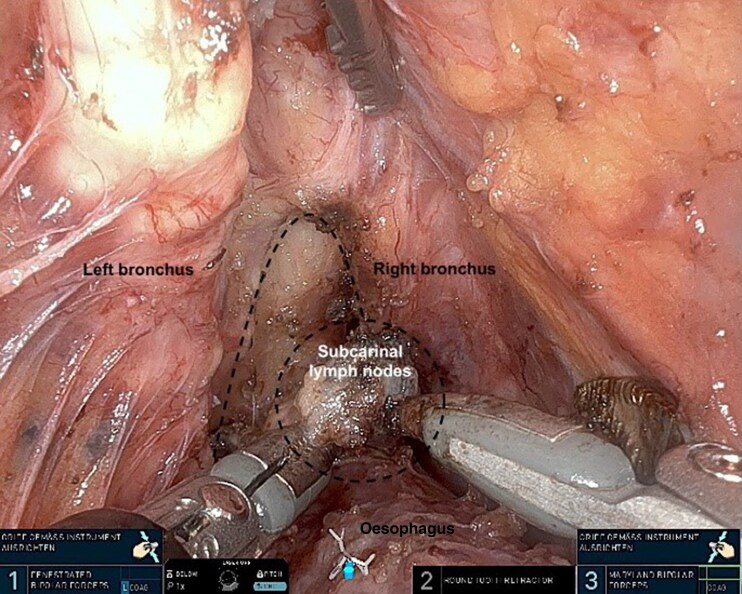
Visualization and dissection of the subcarinal (infracarinal) lymph nodes The dashed lines mark the tracheal bifurcation and subcarinal (infracarinal) lymph nodes.

The procedure was performed as described previously^10,18,19^. The abdominal and the cervical phase can be performed simultaneously or sequentially. The SP RACE procedure was performed sequentially in 18 patients, with the abdominal part performed laparoscopically as the first step followed by the TC robotic SP part, and simultaneously in the remaining two patients, with the laparoscopic and robotic parts performed concurrently using a two-team approach. The visualization and dissection of the left recurrent laryngeal lymph nodes, the thoracic duct (with and without indocyanine green fluorescence in Firefly mode), and the subcarinal (infracarinal) lymph nodes are shown in *Figs 2–5*, respectively. The key procedural steps are presented in *Video S1*.

### Statistical analysis

Statistical analyses were performed using SPSS (Version 30.0.0; IBM Corp., Armonk, NY, USA). Continuous variables are presented as the mean(standard deviation (s.d.)) or median (range), depending on their distribution. Categorical variables are presented as frequencies and percentages. Comparisons between groups were performed using Fisher's exact test for categorical variables and the Mann–Whitney *U* test or Student's *t* test for continuous variables, as appropriate. Two-sided *P* < 0.05 was considered statistically significant. Given the exploratory nature of this study and the limited sample size, all analyses are descriptive and primarily intended to assess trends rather than definitive statistical inference.

## Results

### Patient characteristics

The demographic characteristics of all patients and for those in Groups A and B separately are summarized in *Table 1*. Ten patients (50%) were female, and the median age was 69 (range 49–83) years. The median body mass index of the cohort was 22.6 (range 15.4–35.3) kg/m^2^. Six patients (30%) had adenocarcinoma: two located in the middle oesophagus and four at the gastroesophageal junction (AEG type I, according to the Siewert classification)^15,20^. Fourteen patients (70%) had squamous cell carcinoma: three in the upper oesophagus, nine in the middle oesophagus, and two in the lower oesophagus. Fourteen patients (70%) had undergone neoadjuvant treatment, either chemotherapy according to the FLOT (fluorouracil, leucovorin, oxaliplatin, docetaxel) regimen (with or without immunotherapy) or chemoradiotherapy after the CROSS (chemoradiotherapy for oesophageal cancer followed by surgery study) regimen. All patients included in the study had tumours extending above the carinal bifurcation and/or suspiciously enlarged paratracheal and/or cervical lymph nodes, making them eligible for the SP RACE procedure. According to the American Society of Anesthesiologists (ASA) physical status classification system, 7 patients (35%) were classified as grade II, 12 (60%) were classified as grade III, and 1 (5%) was classified as grade IV. The median CCI was 5 (range 3–11), indicating a moderate to high co-morbidity burden associated with increased perioperative risk and overall mortality in this cohort.

**Table 1:** Patient demographics (*n* = 20) and tumour characteristics

Age (years), median (range)	69 (49–83)
**Sex**	
Male	10 (50%)
Female	10 (50%)
BMI (kg/m^2^), median (range)	22.6 (15.4–35.3)
**ASA physical status classification system**	
Grade II	7 (35%)
Grade III	12 (60%)
Grade IV	1 (5%)
CCI, median (range)	5 (3–11)
**Tumour type and location**	
Adenocarcinoma	6 (30%)
Middle oesophagus	2 (10%)
AEG type I	4 (20%)
Squamous cell carcinoma	14 (70%)
Upper oesophagus	3 (15%)
Middle oesophagus	9 (45%)
Lower oesophagus	2 (10%)
**Neoadjuvant treatment**	
No treatment	6 (30%)
Chemotherapy	4 (20%)

Values are *n* (%) unless otherwise stated. BMI, body mass index; ASA, American Society of Anesthesiologists; CCI, Charlson Comorbidity Index; AEG, adenocarcinoma of the oesophagogastric junction.

### Operative parameters

The mean(s.d.) total procedure duration was 332(88) minutes (min), with a mean mediastinoscopic phase duration of 127(100) min and a mean active console time of 91(48) min during the robotic cervical SP phase (*Table 2*). On average, three instruments were used during this phase (fenestrated bipolar forceps, round tooth retractor, and Maryland bipolar forceps), with an average of two instrument exchanges. Total procedure duration, duration of the mediastinoscopic phase, and active console time were numerically slightly lower in Group B but without statistical significance (*Table 2*).

**Table 2:** Perioperative characteristics and postoperative outcomes in all patients and Groups A and B separately

	All patients (*n* = 20)	Group A (*n* = 10)	Group B (*n* = 10)	P*
Total procedure duration (min), mean(s.d.)	332 (88)	367 (92)	297 (72)	0.075
Duration of the mediastinoscopic phase duration (min), mean(s.d.)	127 (100)	147 (131)	104 (47)	0.371
Active console time (min), mean(s.d.)	91 (48)	95 (60)	86 (32)	0.692
Conversion	0 (0%)	0 (0%)	0 (0%)	
Intraoperative complications	0 (0%)	0 (0%)	0 (0%)	
Intermediate care unit stay (days), median (range)	1 (1–86)	1.5 (1–86)	1 (1–2)	0.182
**Pain management**				
Peridural analgesia	0 (0%)	0 (0%)	0 (0%)	
Paravertebral analgesia	0 (0%)	0 (0%)	0 (0%)	
Patient-controlled analgesia				
POD 1	2 (10%)	0 (0%)	2 (10%)	0.136
POD 3	2 (10%)	1 (5%)	1 (5%)	1
POD 5	0 (0%)	0 (0%)	0 (0%)	
**Complications**	16 (80%)	8 (40%)	8 (40%)	1
Gastrointestinal	5 (25%)	1 (5%)	4 (20%)	0.121
Anastomotic leak	4 (20%)	1 (5%)	3 (15%)	0.264
Delayed gastric emptying	2 (10%)	0 (0%)	2 (10%)	0.136
Pulmonary	8 (40%)	4 (20%)	4 (20%)	1
Pneumonia	5 (25%)	3 (15%)	2 (10%)	0.606
Pleural effusion	7 (35%)	3 (15%)	4 (20%)	0.639
Respiratory insufficiency	4 (20%)	3 (15%)	1 (5%)	0.264
Aspiration	1 (5%)	1 (5%)	0 (0%)	0.305
Cardiac	3 (15%)	3 (15%)	0 (0%)	0.060
Ventricular dysarrhythmia	3 (15%)	3 (15%)	0 (0%)	0.060
Cardiac infarction	1 (5%)	1 (5%)	0 (0%)	0.305
Cardiac arrest	1 (5%)	1 (5%)	0 (0%)	0.305
Thromboembolic	2 (10%)	2 (10%)	0 (0%)	0.136
Pulmonary embolism	2 (10%)	2 (10%)	0 (0%)	0.136
Infection	3 (15%)	0 (0%)	3 (15%)	0.060
Wound infection	3 (15%)	0 (0%)	3 (15%)	0.060
Neurologic/psychiatric	14 (70%)	6 (30%)	8 (40%)	0.329
Recurrent laryngeal nerve palsy	13 (65%)	6 (30%)	7 (35%)	0.639
Type Ia	13 (65%)	6 (30%)	7 (35%)	0.639
Delirium tremens	1 (5%)	0 (0%)	1 (5%)	0.305
**Clavien–Dindo grade**				
< IIIb	9 (45%)	4 (20%)	5 (25%)	0.653
≥ IIIb	7 (35%)	6 (30%)	7 (35%)	0.639
LOS (days), median (range)	8 (5–86)	7 (4–86)	9 (6–47)	0.738
Intraoperative mortality	0 (0%)	0 (0%)	0 (0%)	
In-hospital mortality	2 (10%)	2 (10%)	0 (0%)	0.136
30-day mortality	1 (5%)	1 (5%)	0 (0%)	0.305
90-day mortality	1 (5%)	1 (5%)	0 (0%)	0.305
Intensive care unit readmission	4 (20%)	3 (15%)	1 (5%)	0.264
Radical (R0) resection	18 (90%)	9 (45%)	9 (45%)	1
No. of lymph nodes harvested, mean(s.d.)	31 (7)	28 (7)	34 (7)	0.064

Values are *n* (%) unless otherwise stated. Groups A and B included the first ten and second ten consecutive patients undergoing single-port robotic-assisted cervical oesophagectomy, respectively. *Comparisons between groups were performed using Fisher's exact test for categorical variables and the Mann–Whitney U test or Student's t test for continuous variables, as appropriate. min, minutes; s.d., standard deviation; POD, postoperative day; LOS, length of hospital stay.

### Postoperative complications

No conversions were required during either the abdominal or mediastinoscopic phases of the procedure. No intraoperative complications occurred. Most patients recovered quickly, with a median stay of 1 day in the intermediate care unit. However, six patients required intensive care unit treatment for more than 1 day.

In all, 16 patients (80%) experienced postoperative complications, with 7 (35%) experiencing complications classified as Clavien–Dindo grade IIIb or higher. Five patients (25%) developed pneumonia and seven (35%) developed pleural effusion, leading to respiratory insufficiency in four patients (20%). Anastomotic leak occurred in four patients (20%). All leaks were grade 2 and were managed endoscopically with Eso-SPONGE^®^ (Braun Surgical, Barcelona, Spain), except for one patient who initially received Eso-SPONGE^®^ but subsequently required a VacStent^®^ (Möller Medical, Fulda, Germany). No patient required surgical intervention for the anastomotic leak or any other complication. Recurrent laryngeal nerve paresis (RLNP) occurred in 13 patients (65%), was always left-sided, and was classified as Clavien–Dindo grade Ia. All patients recovered from the palsy during follow-up, with complete recovery observed within 6 months. There were no statistically significant differences in postoperative complications between Group A and Group B (*Table 2*). In-hospital mortality was 10%, with one patient dying within 30 days of surgery because of pulmonary embolism and another patient dying within 90 days due to multiple organ failure caused by sepsis resulting from pneumonia and anastomotic insufficiency (*Table 2*).

### Pain management and patient recovery

Pain management was conducted in an interdisciplinary manner with involvement of the anaesthesiology team. Only three patients (15%) required patient-controlled analgesia, with no need for epidural (peridural analgesia) or paravertebral (intercostal catheter) analgesia. The median hospital length stay was 8 days, and the median intermediate care unit stay was 1 day. Four patients (20%) were readmitted to the intensive care unit after transfer to the general ward because of respiratory insufficiency (three patients) and pulmonary embolism (one patient; *Table 2*).

### Short-term oncological outcomes

Lymph node involvement was confirmed histopathologically in five patients (25%). The mean(s.d.) number of lymph nodes harvested was 31(7). The number of harvested lymph nodes was higher in Group B, although the difference did not reach statistical significance (*Table 2*). A detailed description of lymph node status by station is provided in *Table 3*. Radical (R0) resection was achieved in 18 patients (90%). One patient with distal oesophageal squamous cell carcinoma and one with middle-third adenocarcinoma had circumferential and proximal R1 margins, respectively. The pathological stages for these two patients were T3 N2 and T3 N3, respectively. Both had received neoadjuvant therapy (CROSS or FLOT) without immunotherapy and showed tumour regression grades 4 and 3. No patient achieved a complete response after CROSS, whereas one patient had a complete response following FLOT treatment. No recurrence was observed during follow-up.

**Table 3:** Lymph node stations stratified by preoperative CT scan and postoperative histopathology according to the Japanese classification of oesophageal cancer^15^

LN station (JES classification)	Patients with suspicious preoperative LN	Patients with LN positive on histopathology
Superficial cervical (100 spf)	1 (5%)	0 (0%)
Cervical paraoesophageal (101)	4 (20%)	0 (0%)
Deep cervical (102)	2 (10%)	0 (0%)
Peripharyngeal (103)	8 (40%)	0 (0%)
Supraclavicular (104)	0 (0%)	0 (0%)
Upper mediastinal paraoesophageal (105)	4 (20%)	0 (0%)
Upper paratracheal (106 pre/rec)	13 (65%)	1 (5%)
Lower paratracheal (106 tb)	5 (25%)	0 (0%)
Subcarinal (107)	16 (80%)	2 (10%)
Middle mediastinal paraoesophageal (108)	10 (50%)	1 (5%)
Lower mediastinal paraoesophageal (110)	13 (65%)	1 (5%)
Aortopulmonary window (112 aoP)	6 (30%)	1 (5%)
Pulmonary ligament (112 pul)	0 (0%)	0 (0%)
Paracardial (1/2)	12 (60%)	0 (0%)
Left gastric artery (7)	12 (60%)	1 (5%)
Celiac trunk (9)	15 (75%)	1 (5%)
Splenic artery (11 p)	7 (35%)	1 (5%)
Common hepatic artery (8)	13 (65%)	0 (0%)
Hepatoduodenal ligament (12)	3 (15%)	0 (0%)

Values are *n* (%). LN, lymph nodes; JES, Japan Esophageal Society; spf, superficial; pre, pretracheal; rec, recurrent nerve; tb, tracheobronchial; aoP, para-aortic posterior; pul, pulmonary; p, posterior.

## Discussion

This IDEAL stage 2a/b study reports on the successful implementation of the da Vinci SP system in oncological oesophageal surgery and the first series of da Vinci SP RACE oesophagectomies worldwide. No conversions to open or transthoracic procedures were necessary, and no intraoperative complications or deaths occurred, demonstrating the feasibility and intraoperative safety of this new surgical technique for the treatment of oesophageal cancer.

The rate of major complications (Clavien–Dindo grade ≥ IIIb) encountered during SP RACE implementation was comparable to the complication rates observed during the learning curve phase of transthoracic robotic oesophagectomy^21,22^. Pneumonia and anastomotic leakage are the leading complications after oesophageal surgery^21–25^. In the present study, the rates of these two complications were consistent with those reported in literature. Cervical anastomoses have repeatedly been associated with higher rates of anastomotic leakage compared with intrathoracic anastomoses. This has also been demonstrated in a modern Western multicentre randomized trial, where the overall leak rate was significantly higher for cervical *versus* intrathoracic anastomosis (34.1% *versus* 12.3%) and meta-analyses have showed an RR of anastomotic leak approximately 2.5- to 3-fold higher with cervical anastomosis^24,25^. Intrathoracic anastomoses are also associated with lower rates of RLNP and fewer severe complications. These findings are thought to relate to longer conduit length, increased tension, and less favourable blood supply for cervical reconstructions, which may predispose to impaired healing at the anastomotic site^24^. The incidence of RLNP is a quality benchmark^26^. RLNP is of particular importance, because morbid patients with a cervical anastomosis are at increased risk of aspiration and have limited tolerance for chronic microaspiration, emphasizing the need for meticulous nerve preservation. The high incidence of RLNP observed in the present study is most likely attributable, in part, to the institutional policy at the University Hospital Mainz of routine postoperative laryngoscopy, which enables the detection of even clinically subtle nerve dysfunctions that may remain unrecognized when relying solely on symptomatic assessment. Importantly, all cases were classified as type Ia left-sided RLNP and required only conservative logopaedic therapy. No patient developed permanent RLNP during follow-up, indicating a transient and clinically limited impairment rather than a relevant surgical complication. Furthermore, it is believed that the low pneumonia rate observed in the present cohort may be explained by the proactive perioperative management strategy, including preoperative and/or postoperative endoscopic pyloric balloon dilatation. This approach likely reduced delayed gastric emptying and subsequent aspiration risk, thereby positively influencing pulmonary outcomes.

SP RACE avoids a thoracotomy, which is one of the key factors driving prolonged hospital recovery after McKeown or Ivor Lewis approaches. The reported length of hospital stay in the present study compares favourably with existing evidence, supporting the claim that SP RACE can result in enhanced recovery in select patients. No patient required epidural or paravertebral analgesia. Only two patients required patient-controlled analgesia on postoperative day 3, whereas all others were treated with oral or intravenous analgesics on demand. This resulted in very fast mobilization of the patients, early transfer to the normal ward, and discharge for patients without complications, the earliest being on postoperative day 5. These results are consistent with, or slightly better than, previous reports on SP and multiport Ivor Lewis oesophagectomy^23,27^. Compared with minimally invasive cervical oesophagectomy and McKeown procedures, the length of hospital stay was similar or slightly shorter in the present study^28–31^. However, it should be noted that because this is a novel and technically demanding approach, the outcomes reported here are preliminary and warrant further validation.

The high intensive care unit readmission rate was due to complications, such as respiratory insufficiency following pulmonary infection, anastomotic leakage, and pulmonary embolism. Most of the patients recovered and were successfully discharged after the treatment of complications. However, one patient died 4 days after the procedure due to a fulminant pulmonary embolism, and another died in the intensive care unit 86 days after surgery from multiple organ failure caused by sepsis resulting from pneumonia and anastomotic insufficiency. Whether the pulmonary embolism was related to the pneumomediastinum remains unclear, but both patients had a high CCI (5 for the patient who died 4 days after the procedure and 11 for the patient who died 86 days after surgery) reflecting their multimorbidity.

The mean lymph node yield in this series surpassed the benchmark recommendations^32^. The value of extensive three-field lymphadenectomy in oesophageal cancer remains controversial. Recent studies comparing three-field and two-field lymphadenectomy in patients with middle and lower thoracic oesophageal cancer found no improvement in overall survival or disease-free survival with the addition of cervical lymph node dissection, despite a higher lymph node yield in the three-field group^33,34^. A systematic review and meta-analysis has not conclusively demonstrated a survival benefit from prophylactic cervical lymphadenectomy and has highlighted increased morbidity associated with more extensive dissection^35^. These findings raise important questions about the routine need for a potentially more morbid approach, particularly in the absence of clear survival advantages, and support an individually tailored strategy to lymphadenectomy in oesophageal cancer surgery.

Interestingly, in the present study, no patient achieved a complete response after CROSS, whereas one patient had a complete response following FLOT treatment. The rate of radical R0 resections may have been influenced by the learning curve effect observed in this study, as well as in other studies presenting pioneering surgical approaches^36,37^. Although analysis of the learning curve was beyond the scope of this study, no significant differences were observed in any of the parameters analysed between the first (Group A) and second (Group B) half of patients. The yield of harvested lymph nodes was higher, albeit not significantly, in Group B. In addition, despite the lack of significant differences, total procedure duration, duration of the mediastinoscopic phase, and active console time were lower in Group B. These observations suggest that familiarity and confidence with the procedure increased over time. Several studies in the literature have reported on the operative times for oesophagectomies. Biere *et al*. measured an average duration of 299 min for open oesophagectomy compared with 329 min for minimally invasive oesophagectomy^5^. Van der Sluis *et al*. investigated the learning curve of three-stage RAMIE in a cohort of 312 patients and, after the completion of the learning curve, found a mean operative time of 375 min^21^. In the present cohort, the total procedure duration was 332 min, which is shorter than published data on SP RACE, especially when sequential procedures are considered^38–41^. Improved results could potentially be achieved by overlapping the transabdominal and TC SP phases to allow simultaneous procedures, a strategy demonstrated by Grimminger *et al*. and analysed further by Fujita *et al*.^8,41^. In those studies, this approach significantly reduced total procedural time to approximately 150 min.

The present study has several limitations. The decision not to directly compare the SP RACE patient cohort with patients undergoing multiport robot-assisted oesophagectomy was primarily due to the limited number of SP RACE procedures performed to date. Perioperative and postoperative data, particularly complication rates, may have been influenced by the steep learning curve associated with the SP RACE technique. Fluoroscopic swallowing assessment was not routinely performed in patients with RLNP. This represents a limitation of the present study, and the added diagnostic value and clinical impact of this modality warrant evaluation in future studies. In addition, the recent adoption of the da Vinci SP RACE platform and the relatively short follow-up period prevented evaluation of its impact on overall survival and disease recurrence. Therefore, future studies are needed to validate the findings of the present study, to standardize operative protocols, to refine surgical techniques, and to define appropriate indications for SP RACE. Nonetheless, the present study demonstrates that SP RACE can be successfully integrated into high-volume centres, expanding surgical options for patients with proximal oesophageal tumours and significant co-morbidities.

TC mediastinal lymphadenectomy procedures such as SP RACE may have a future role in highly selected patients, particularly when conventional operative approaches and organ-preserving strategies, such as sentinel node and neoadjuvant therapy-oriented (SANO) surveillance, have been ruled out. The recent phase III SANO trial compared active surveillance with standard oesophagectomy in patients with a clinical complete response after neoadjuvant chemoradiotherapy and demonstrated that overall survival at 2 years with active surveillance was non-inferior to immediate surgery (74% *versus* 71%, respectively) in a multicentre stepped-wedge cluster-randomized design, with similar rates of postoperative complications and mortality between groups^42^. These findings reinforce the concept that select organ-preserving strategies may be appropriate in specific subgroups and underscore that more morbid approaches, such as routine three-field lymphadenectomy, should be reserved for patients with confirmed malignant upper mediastinal nodes or when all other options are excluded. This is particularly relevant in Western populations, where such presentations are uncommon and the survival benefit of extensive lymphadenectomy remains unproven.

SP RACE may offer a surgical option for a highly selected patient population, particularly those with pulmonary co-morbidities who may otherwise be considered unsuitable for surgery, by avoiding a transthoracic approach. The technique demonstrated short operating times, rapid postoperative recovery, and adequate lymph node yield, and postoperative pain was minimal even without epidural or paravertebral analgesia. However, the scope of the SP RACE procedure currently remains limited, and its outcomes warrant further prospective investigation to better define its role, refine patient selection, and evaluate long-term results.

## Supplementary Material

zrag030_Supplementary_Data

## Data Availability

The data sets used and analysed in this study are available from the corresponding author upon reasonable request.
