# Supplementary figures and images for: First series of da Vinci single-port robotic-assisted cervical oesophagectomy: single-centre IDEAL stage 2a/2b study
Source: BJS Open. 2026 Jun 5;10(3):zrag030. doi: 10.1093/bjsopen/zrag030 (PMC13237595; doi:10.1093/bjsopen/zrag030)

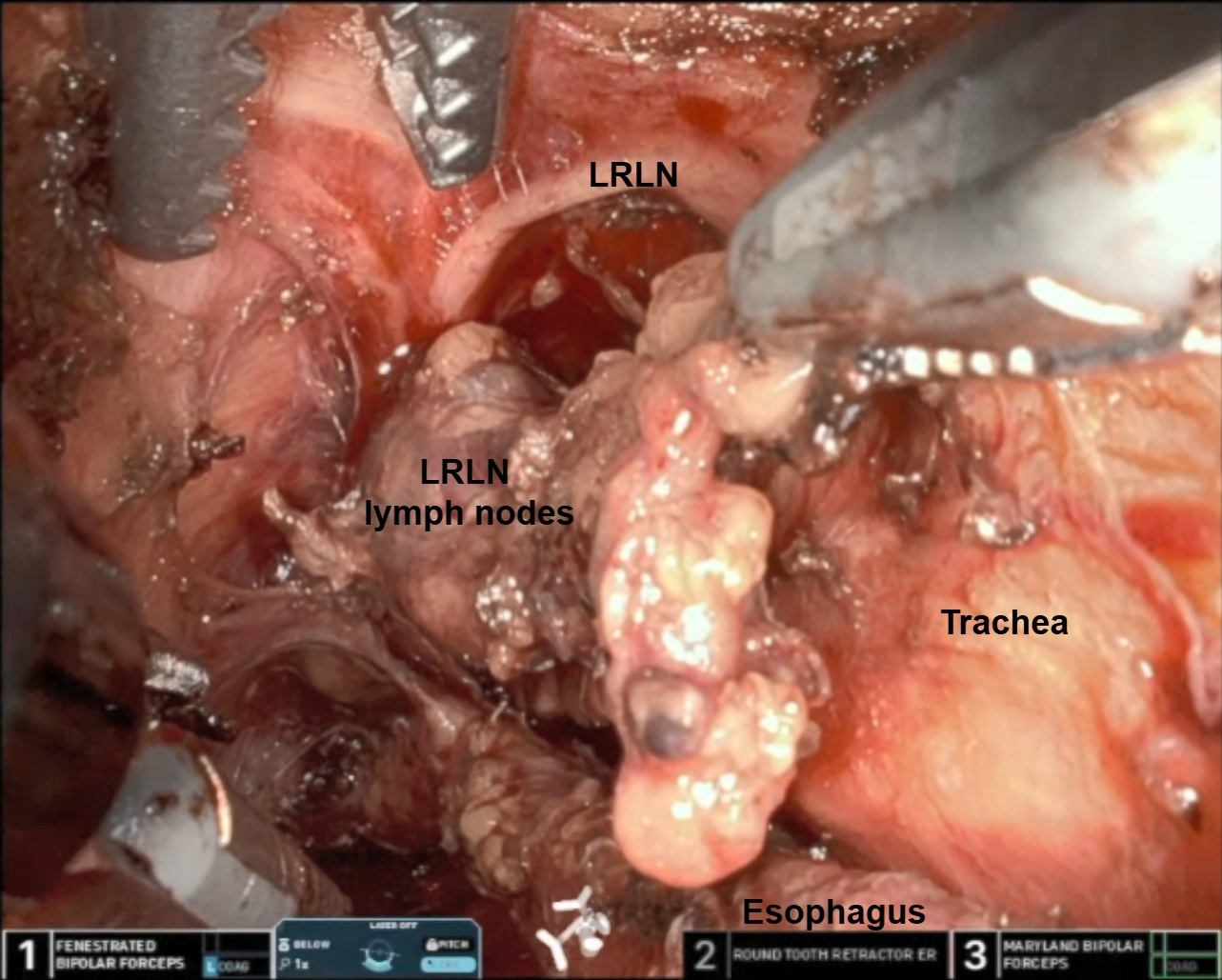

Supplement: zrag030_Supplementary_Data [file zrag030_supplementary_data.zip › Video_still_1.jpg]

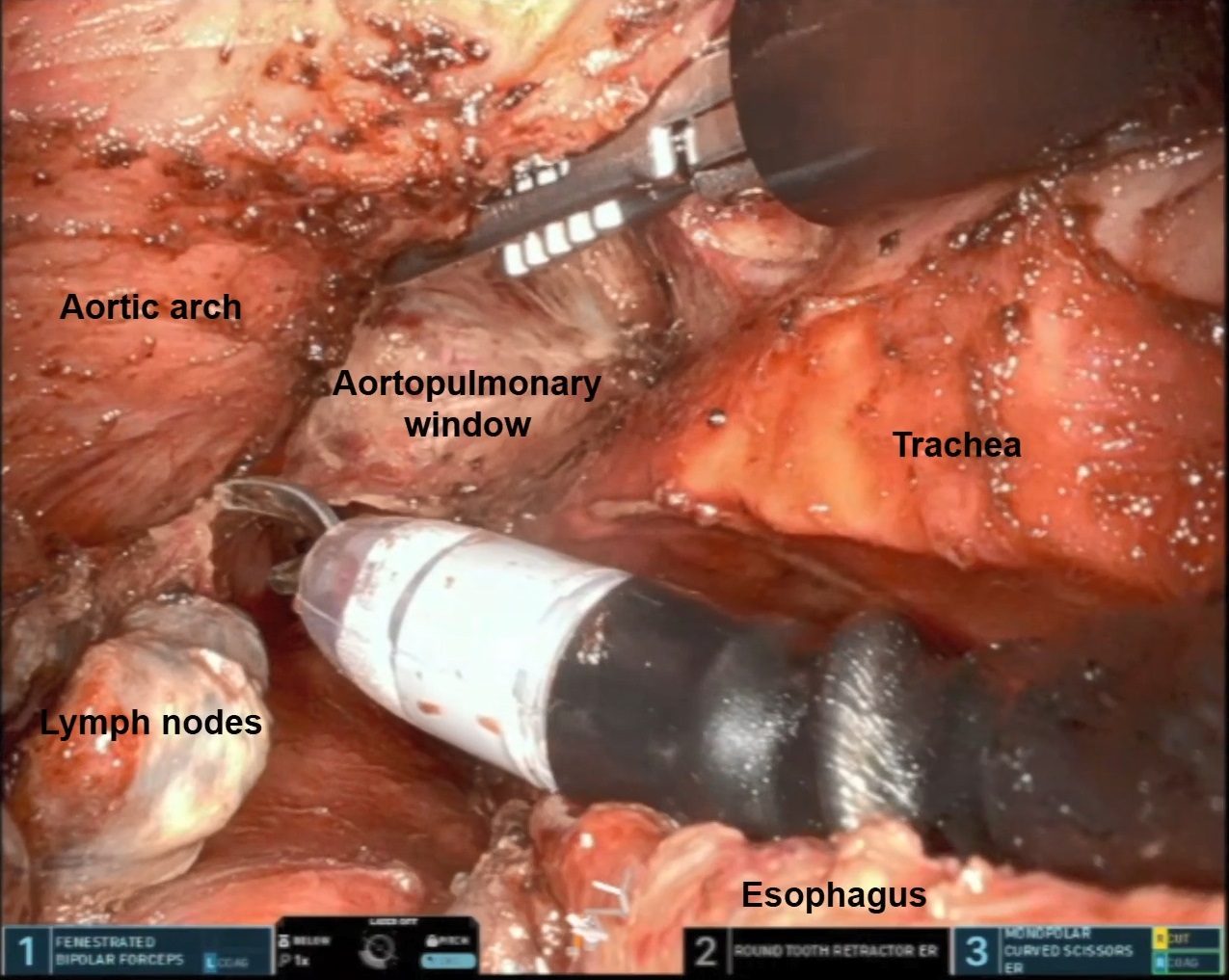

Supplement: zrag030_Supplementary_Data [file zrag030_supplementary_data.zip › Video_still_2.jpg]

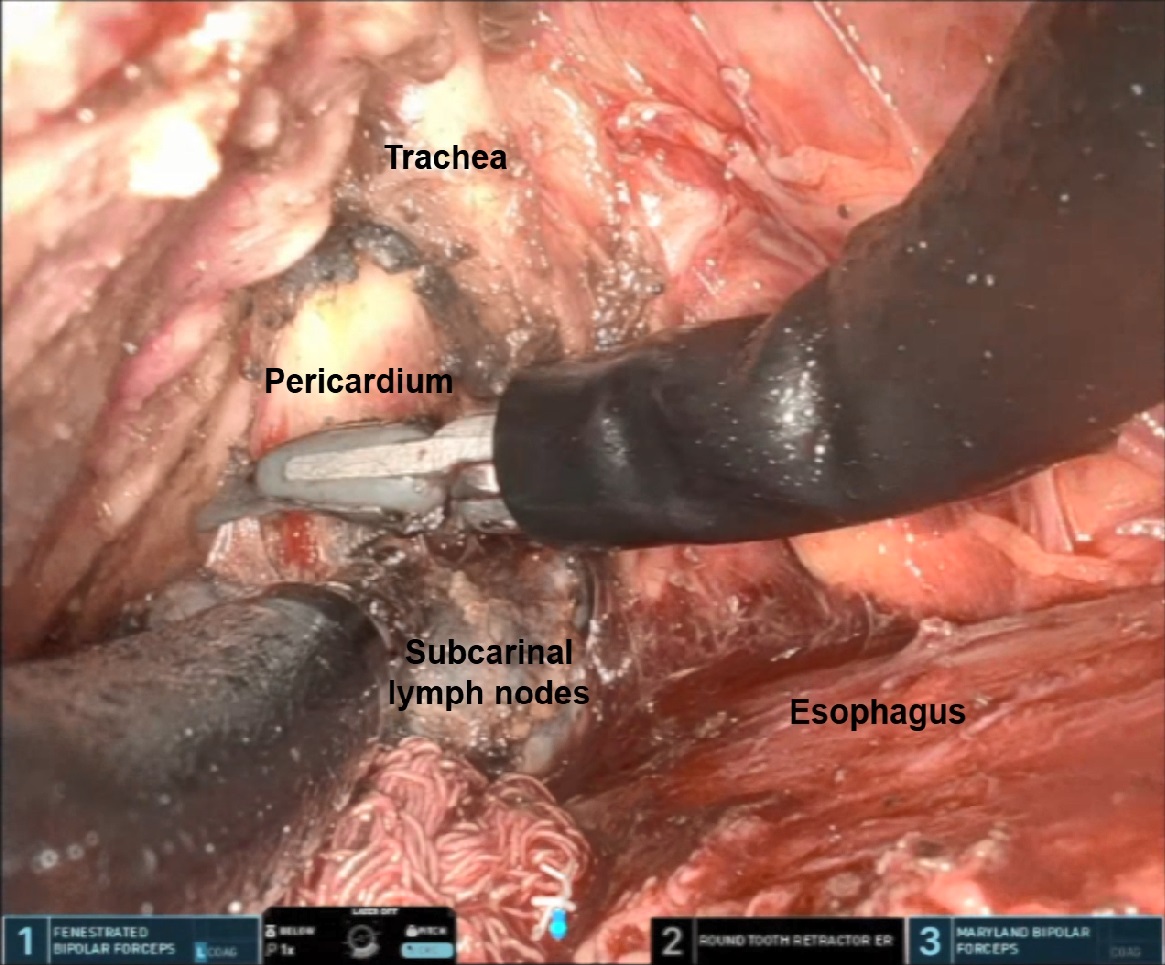

Supplement: zrag030_Supplementary_Data [file zrag030_supplementary_data.zip › Video_still_3.jpg]
